# Propensity score analysis using the freely available user-friendly software EZR (Easy R)

**DOI:** 10.1007/s44313-026-00122-9

**Published:** 2026-01-22

**Authors:** Yoshinobu Kanda

**Affiliations:** Division of Hematology, Jichi Medical University, 3311-1, Yakushiji, Shimotuske-shi, Tochigi 329-0498 Japan

**Keywords:** Propensity score, Statistical software, Matching, Inverse probability weighting, EZR

## Abstract

EZR (Easy R) is a statistical software package that is freely available on our website (https://www.jichi.ac.jp/usr/hema/EZR/statmed.html) and can be used on both Windows (Microsoft Corporation, USA) and macOS (Apple, USA) systems. EZR is built on R and R Commander and offers a range of statistical functions, including survival analyses with competing risks or time-dependent covariates, receiver operating characteristic curve analyses, meta-analyses, and sample size calculations, all accessible through a point-and-click graphical interface. A previous report that described the installation and basic operation of EZR (“Investigation of the freely available easy-to-use software ‘EZR’ for medical statistics”, *Bone Marrow Transplant*, 2013) has been cited in more than 14,000 scientific papers as of November 2025. This report describes the procedures for performing propensity score (PS) analysis, including PS matching and inverse probability weighting, and compares these approaches with conventional multivariate analyses.

## Introduction

Evidence-based medicine (EBM) involves interpreting findings from clinical research, that is, the available evidence, and applying those findings carefully when planning treatment for individual patients, taking into account their clinical conditions and personal preferences. Therefore, further clinical research aimed at generating additional evidence is continuously necessary. In this sense, EBM and clinical research are inseparably linked, as both originate from clinical questions that arise in daily practice. Within this framework, a basic understanding of statistics is essential not only for evaluating the validity of existing studies but also for conducting one’s own research. Even when biostatisticians are available for collaboration, clinicians still require a fundamental level of statistical knowledge to conduct their studies effectively.

Consequently, there are inevitable situations in which clinicians must use statistical software themselves. However, commercially available statistical programs are often expensive for individual users, and their command structures can be difficult to learn. In contrast, R is freely available; however, performing analyses by entering scripts is difficult for clinicians [1]. By integrating the R Commander into R, data analysis can be performed using mouse-based operations alone [2]; however, the statistical functions available by default are limited.

Therefore, I developed and released a customized version of R Commander called EZR (Easy R), which incorporates a wide range of statistical analysis functions. EZR is free to use (available at https://www.jichi.ac.jp/usr/hema/EZR/statmed.html) and enables advanced statistical analyses, such as competing risk analysis and analyses with time-dependent covariates, through simple graphical operations. In addition, EZR was designed with academic presentations and publications in mind, allowing users to automatically generate formatted patient background tables and analyze the results for presentation slides or manuscript submissions. Moreover, because R scripts generated automatically through mouse operations can be saved, users can learn R scripting, document analytical procedures, and enable supervisors to review their analyses.

A previous report that provided instructions for installing and using EZR has been cited in more than 14,000 scientific papers as of November 2025 [3]. Since the publication of this paper, several important statistical functions have been added, including restricted mean survival time, propensity score (PS), current leukemia-free survival, and network meta-analyses. However, EZR is distributed with “absolutely no warranty,” just like R itself, and the conditions for redistribution follow those for R and R Commander under the GNU General Public License. In the present report, I introduce a method for performing PS analyses, including PS matching and inverse probability weighting.

### Installing EZR

The process for installing EZR has been described in a previous report [3]. Briefly, for Windows users, the only required file is EZRsetupENG.exe, which can be downloaded from our website. EZR is installed alongside R and R Commander simply by running this installer on Windows (XP, VISTA, 7, 8, 10, and 11). The default data folder is “C:\EZRDATA”, which can be changed by right-clicking the shortcut, selecting “Properties,” and modifying the folder name in the “Target:” field on the “Shortcut” tab.

Alternatively, EZR can be installed in three steps: installing R, installing the R Commander package, and installing the EZR plugin package. This method allows users to work with the latest version of R, but it may result in errors due to conflicts among the updated packages required by EZR. Users of Mac OS X must use this alternative method. Detailed instructions are available on our website under the sections “EZR for Windows” and “EZR for macOS”.

### Basic EZR operation

The EZR operations have been described in detail in a previous study [3]. Briefly, EZR can be started by using the shortcut on the desktop or by selecting EZR from the “Start” menu. When launched, two windows appear on the desktop: one titled “R Console” and the other titled “R Commander”, the latter serving as the main working window for EZR.

EZR functions can be accessed from a menu bar located immediately below the title bar. EZR automatically generates and executes the corresponding R commands, which are displayed in the “R Script” window. The results appear in the “Output” window, and any errors or warnings are shown in the “Messages” window.

The datasets saved as Excel files (.xls or .xlsx) or comma-separated values (CSV) files (.csv) can be imported into EZR by selecting “File” > “Import data” > “From Excel, Access or dBase data set” or “File” > “Import data” > “Read Text Data From File, Clipboard, or URL,” respectively. Alternatively, the data can be imported using a copy-and-paste approach. Data of interest copied from a spreadsheet, text file, website, or other sources can be imported into EZR by selecting “File” > “Import data” > “Read Text Data From File, Clipboard, or URL.” When pasting from the clipboard, users should select “Clipboard” for “Location of Data file” and “Tabs” for “Field Separator” in the dialogue. EZR can also import SPSS and Stata data files.

**Fig. 1:**
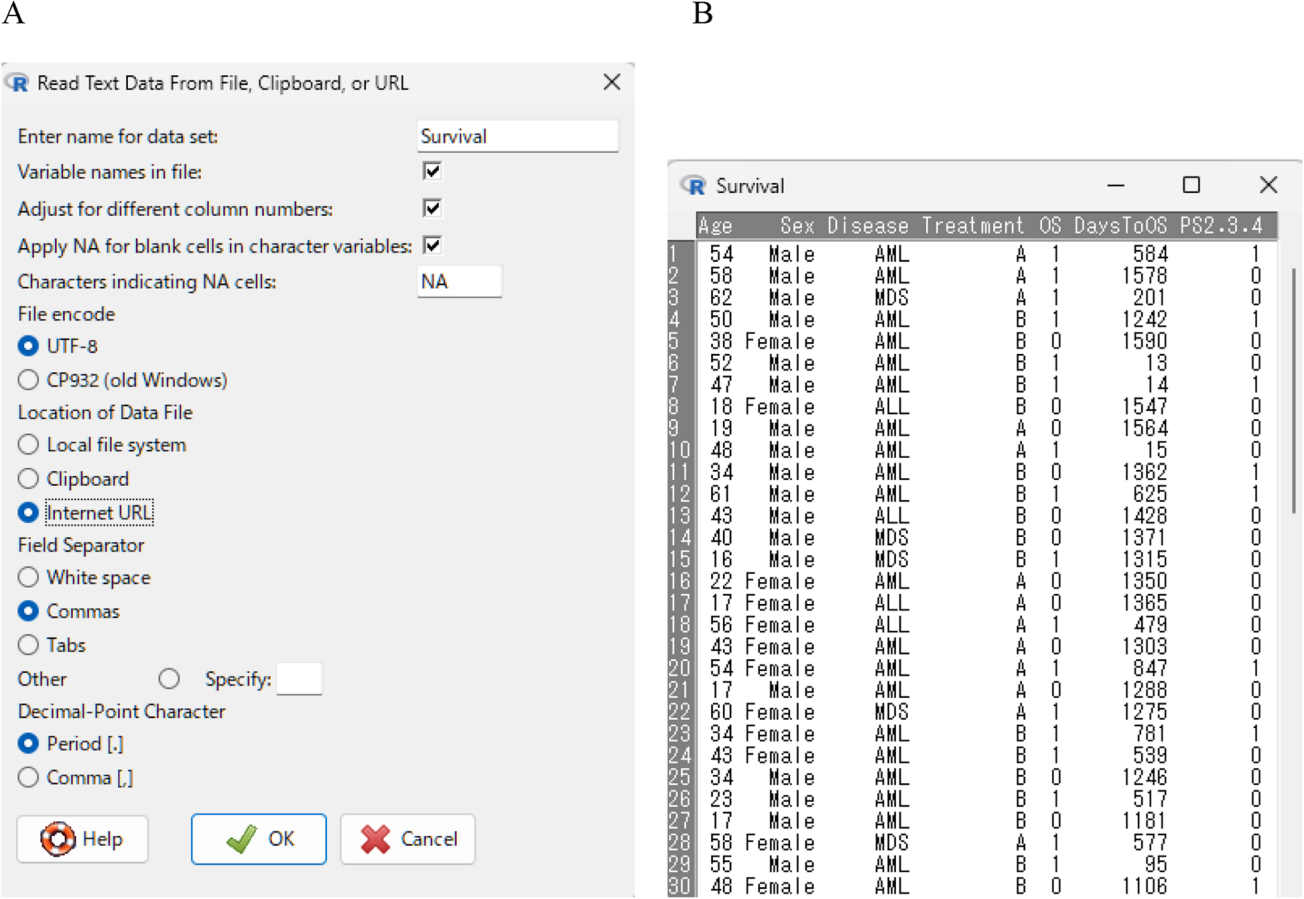
**A** Dialogue box for importing text data into EZR. **B** The dataset “Survival” after it has been imported into EZR

In the following instructions, a sample dataset containing 74 fictional patients who received treatment A or B for hematological malignancies will be used. The data file “Survival.csv” is available at https://www.jichi.ac.jp/usr/hema/EZR/file/Survival.csv. Users can import the file directly into EZR by selecting “Internet URL” for “Location of Data File” after choosing “File” > “Import data” > “Read Text Data From File, Clipboard, or URL”. The dataset name used in R can be freely specified by entering the desired name (“Survival” in this example) in the “Enter name for data set:” field (Fig. 1A).

Imported data can be viewed by clicking the “View data set” button (Fig. 1B) and edited directly by clicking the “Edit data set” button. Users can create new variables or modify existing ones using the functions under “Active data set” on the menu bar. The modified dataset can be saved as an R file (.rda) by clicking the “Save” button or selecting “File” > “Active data set” > “Save active data set”. Only the active dataset shown to the right of “Data set:” is saved. The saved dataset can be reloaded by selecting “File” > “Load data set.”

### Summarizing patient characteristics

When researchers aim to evaluate the effect of a treatment, the ideal approach is to conduct a prospective randomized clinical trial in which participants are randomly assigned to either the treatment or control groups. However, these trials are often difficult to conduct. Observational studies play an important role in providing clinical evidence. A major limitation of observational studies is confounding bias. For example, in a comparison between intensive and less intensive treatments, younger and fitter patients are more likely to receive intensive therapy, potentially leading to biased outcome estimates. Therefore, summarizing patient characteristics is a critical first step.

A table that summarizes patient characteristics can be created by selecting “Graphs and tables” > “Summary table of sample characteristics.” In this case, the grouping variable “Treatment” should be specified in the “Grouping variable” box, and categorical variables such as “Disease,” “PS2,3,4” (coded as 0 for ECOG performance status 0–1, and 1 for 2–4), and “Sex” should be specified in the “Categorical variable” box. Multiple variables can be selected by clicking while holding the “Ctrl” key. Continuous variables should be specified in the “Continuous variables (normal distribution)” box or the “Continuous variables (non-normal distribution)” box, depending on the distribution of each variable. If the “Show standardized differences” option is selected, standardized differences will be displayed as balance diagnostics; values less than 0.10 are commonly used as a threshold to indicate adequate balance. In the present example, all four background characteristic variables were imbalanced, with standardized differences exceeding 0.10 (Table 1A). Notably, the median age was 55 years in patients receiving treatment A, compared with 38 years in those receiving treatment B, which may have strongly influenced the comparison between the two groups. Multivariate analyses are often used to account for background differences.

**Table 1:**
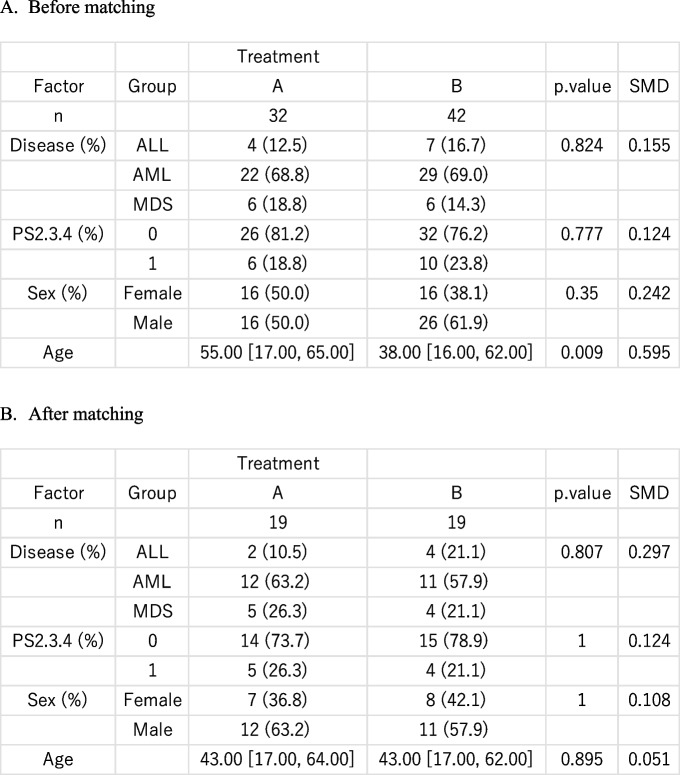
Characteristics of the patients before and after propensity score matching

*SMD* standardized mean difference

### Cox proportional hazard modeling for survival analysis

**Fig. 2:**
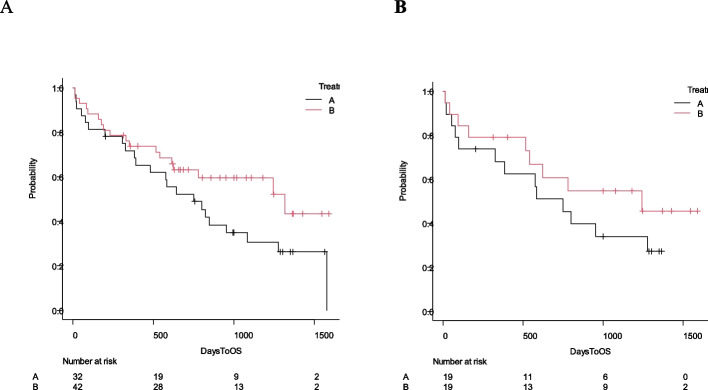
Kaplan–Meier curves before (**A**) and after (**B**) propensity score matching

A survival analysis can be performed by selecting statistical functions in the “Statistical analysis” > “Survival analysis” menu. Kaplan–Meier curves and comparisons of survival curves using the log-rank test can be produced by selecting “Statistical analysis” > “Survival analysis” > “Kaplan–Meier survival curve and log-rank test”. At least two variables are required: a time-to-event variable, which represents the time to the occurrence of an event (death in survival analysis) or the time to the last follow-up for patients without an event, and a status variable, coded as 1 for an event and 0 for no event. In this example, “DaysToOS” should be selected in the “Time-to-event variable” box and “OS” in the “Status indicator” box. To compare survival curves between treatment A and treatment B, “Treatment” should be selected in the “Grouping variable” box. The results of the log-rank test will appear in the “Output” window, and a Kaplan–Meier survival curve appears in a separate window (Fig. 2A).

For multivariate analysis of survival data, a Cox proportional hazards regression analysis can be performed by selecting “Statistical analysis” > “Survival analysis” > “Cox proportional hazard regression,” followed by specifying “DaysToOS” as the “Time” variable, “OS” as the “Event” variable, and “Age,” “Disease,” “PS2,3,4,” “Sex,” and “Treatment” as the “Explanatory variables” by double-clicking in the “Variables” box. In the “Output” window, the main results of the Cox proportional hazards regression analysis will be displayed, including hazard ratios, their 95% confidence intervals, and *p*-values for each explanatory variable. In this analysis, the effect of treatment ([T.B] indicates the effect of treatment B compared with treatment A) was not statistically significant after adjusting for background characteristics, with a hazard ratio of 0.64 and a *p*-value of 0.21 (Table 2A). More detailed methods for EZR survival analysis have been described previously [3].

**Table 2:**
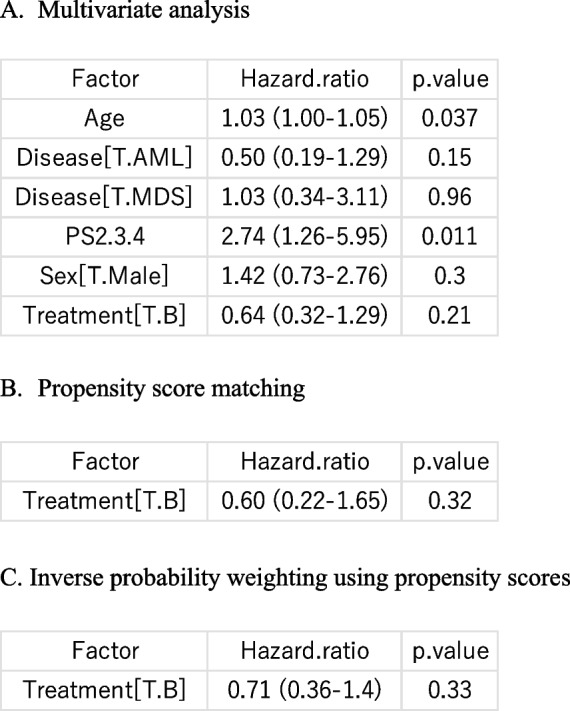
The effect of treatment on survival was assessed using different methods to adjust for background characteristics

### Creating a PS variable

PSs have become widely used in recent years to reduce bias by balancing background characteristics between groups [4]. In clinical practice, treatment decisions are typically based on multiple factors. To account for this, a logistic regression model is constructed in which the treatment status is the dependent variable and background characteristics are the independent variables. This model estimates the probability of each patient receiving a given treatment, defined as the PS. The PS can then be used for matching, stratification, covariate adjustment, and inverse probability weighting (IPW). Compared with standard multivariate analyses, the PS offers advantages, such as allowing adjustment for many confounders, even in studies with relatively small sample sizes, and enabling matching based on the score so that the resulting comparison resembles a quasi-randomized design with an improved balance between groups.

In the current analysis, the “Treatment” variable cannot be used directly in a logistic regression model to create a PS, because the dependent variable in logistic regression must be coded as 0 and 1, whereas the “Treatment” variable has the values “A” and “B.” To resolve this, a new variable, “Treatment01,” can be created by selecting “Active data set” > “Variables” > “Create new variable”, entering “Treatment01” in the “New variable name” field, and entering “ifelse(Treatment = = ”A,” 0, 1)” in the “Expression to compute” field. The “ifelse(*test*, *yes*, *no*)” function returns one of two specified values, *yes* or *no*, depending on whether the condition in *test* is true or false. Thus, “ifelse(Treatment = = ”A,” 0, 1)” returns 0 for patients in the treatment A group and 1 for those in the treatment B group.

**Fig. 3:**
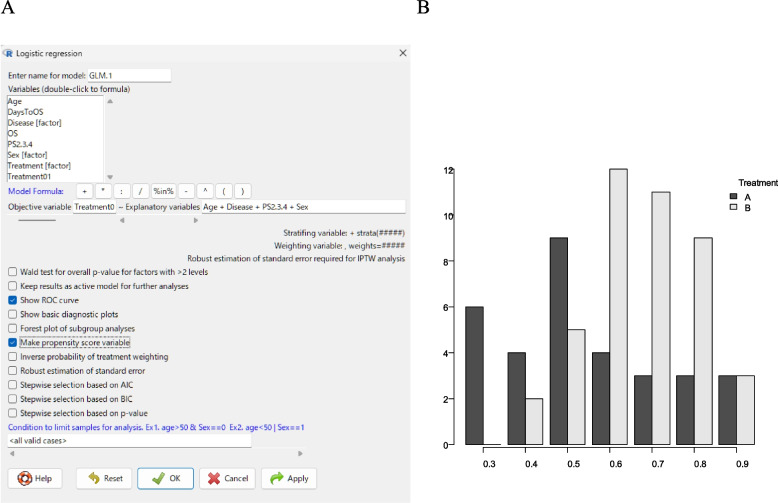
**A** Dialogue box for creating a propensity score variable. **B** Histogram showing the distribution of propensity scores by treatment group

A PS variable can be created by selecting “Statistical analysis” > “Discrete variables” > “Logistic regression,” followed by specifying “Treatment01” as the “Objective variable” field and “Age,” “Disease,” “PS2,3,4,” and “Sex” as the “Explanatory variables” field by double-clicking in the “Variables” box (Fig. 3A). By selecting the “Make propensity score variable” option, a new PS variable named PropensityScore.GLM.X will be generated (the suffix GLM.X varies depending on the model name used). This score represents the estimated probability of receiving Treatment B. If the “Show ROC curve” option is selected, a receiver operating characteristic (ROC) curve will be displayed, and the area under the ROC curve (AUC) will appear in the “Output” window. When the AUC is between 0.6 and 0.9, the PS is generally considered useful for adjusting for background differences. In the present study, the AUC was 0.696, indicating a reasonable level of predictive accuracy. When the AUC is excessively high (≥ 0.9), the two groups become almost perfectly separated by the PS. In such cases, only a small number of matched pairs are formed, making subsequent matching analyses impractical.

A histogram of the PS is also useful for evaluating whether the generated PS functions appropriately. It can be created by selecting “Graphs and tables” > “Histogram.” By specifying “PropensityScore.GLM.X” in the “Variable” box and “Treatment” in the “Grouping variable” box, a histogram will be displayed (Fig. 3B). As expected, patients in the Treatment B group tended to have higher PSs, whereas those in the Treatment A group tended to have lower PSs. However, the histogram also indicates that only a small subset of patients was suitable for matching based on the PS.

### PS matching

When performing PS matching, caliper matching is commonly used. In this approach, matched pairs are required to have PSs that differ by no more than 0.2 times the standard deviation of the logit of the scores [5]. However, a caliper defined as 0.2 times the standard deviation of the PS itself is also used [6], and in many situations, it yields similar matching results. In this instruction, the latter caliper setting is used to simplify the procedure.

The matching function in EZR can be found under “Statistical analysis” > “Matched-pair analysis” > “Extract matched controls”. If “Treatment01” is selected as the “Grouping variable” and “PropensityScore.GLM.X” as the “Matching variables,” and the “Caliper matching” option is set to “Yes,” a new dataset named Survival_MP is created. In this example, the original dataset included 32 and 42 samples in the Treatment A and B groups, respectively; however, after matching, only 19 pairs were retained in the Survival_MP dataset, as shown in the “Output” window.

It is important to assess whether the matching procedure adequately balances the background characteristics. This can be checked by selecting “Graphs and tables” > “Summary table of sample characteristics.” As shown in Table 1B, the standardized differences for “Age” improved to < 0.10, whereas the balance for “Disease,” “PS2.3.4,” and “Sex” remained insufficient, with values exceeding 0.10.

After matching, the survival curves of the two groups were more similar than before matching (Fig. 2B). By selecting “Statistical analysis” > “Matched-pair analysis” > “Stratified Cox proportional hazard regression for matched-pair analysis”, a Cox proportional hazard modeling stratified by matched pairs can be performed using “Treatment” as the only explanatory variable and “pairmatch” as the stratifying variable. This analysis yielded a hazard ratio of 0.60 with a *p*-value of 0.32. Although stratified Cox proportional hazards regression is recommended for matched-pair analysis, conventional Cox proportional hazards regression is commonly used in the literature [7].

After completing the analyses using the matched dataset, the active dataset should be switched back to the original dataset to perform additional analyses, including the following inverse probability weighting analysis: In EZR, this can be done by clicking the box next to “Data set:” located below the menu bar, which allows the user to select the active dataset. In this case, “Survival” should be selected.

### IPW using the PS

A major drawback of the matching procedure is that unmatched samples are discarded, which reduces statistical power. In addition, individuals with extremely high or low PSs were excluded during matching, resulting in an analytical dataset whose distribution differed from that of the original population.

IPW addresses these limitations by performing regression analyses in which weights are defined as the inverse of the PS (in the Treatment B group in this example) or one minus the PS (in the Treatment A group). These weights increase the influence of strata with fewer patients. When IPW is applied, the analysis differs depending on the estimate: the average treatment effect (ATE) or the average treatment effect on the treated (ATT). The ATE represents the expected difference if all individuals are moved from the untreated to the treated condition (from Treatment A to Treatment B in this case), whereas the ATT represents the expected difference if only untreated individuals (those receiving Treatment A) are moved to the treated condition (Treatment B). Although the weighting schemes differ between the ATE and the ATT, the ATE is more commonly used in IPW analyses; therefore, the ATE is used as the default in EZR. Under this approach, individuals in the Treatment B group with low PSs receive higher weights, and those in the Treatment A group with high PSs also receive higher weights. This process improves the PS balance between the two groups.

However, when the PS was 0.1, the corresponding weight becomes 10, indicating that the individual was treated as equivalent to 10 people in the analysis. Stabilized weights are often applied to reduce this type of instability. Stabilized weighting multiplies each individual’s inverse probability weight by the proportion of the total sample belonging to that individual’s treatment group. For instance, if the Treatment B group accounts for 40% of the sample and the Treatment A group accounts for 60%, the weights for individuals in the Treatment B group were multiplied by 0.4, and those for individuals in the Treatment A group were multiplied by 0.6.

To assess the covariate balance after weighting, standardized differences were calculated using weighted data. However, when calculating the standardized differences for categorical variables with three or more categories, EZR requires the values to be numeric rather than character strings such as “AML,” “ALL,” or “MDS” in the “Disease” variable. Therefore, a new variable named “Disease012” should be created before performing the weighting procedure. This can be done by selecting “Active data set” > “Variables” > “Create new variable”, entering “Disease012” as the “New variable name,” and entering the expression “ifelse(Disease = = "ALL, “0, ifelse(Disease = = "AML, “1, 2))” in the “Expression to compute” field. This new variable must be treated as a categorical variable rather than a continuous variable. The variable type can be changed by selecting “Active data set” > “Variables” > “Convert numeric variables to factors” and selecting “Disease012” in the “Variable” box.

**Fig. 4:**
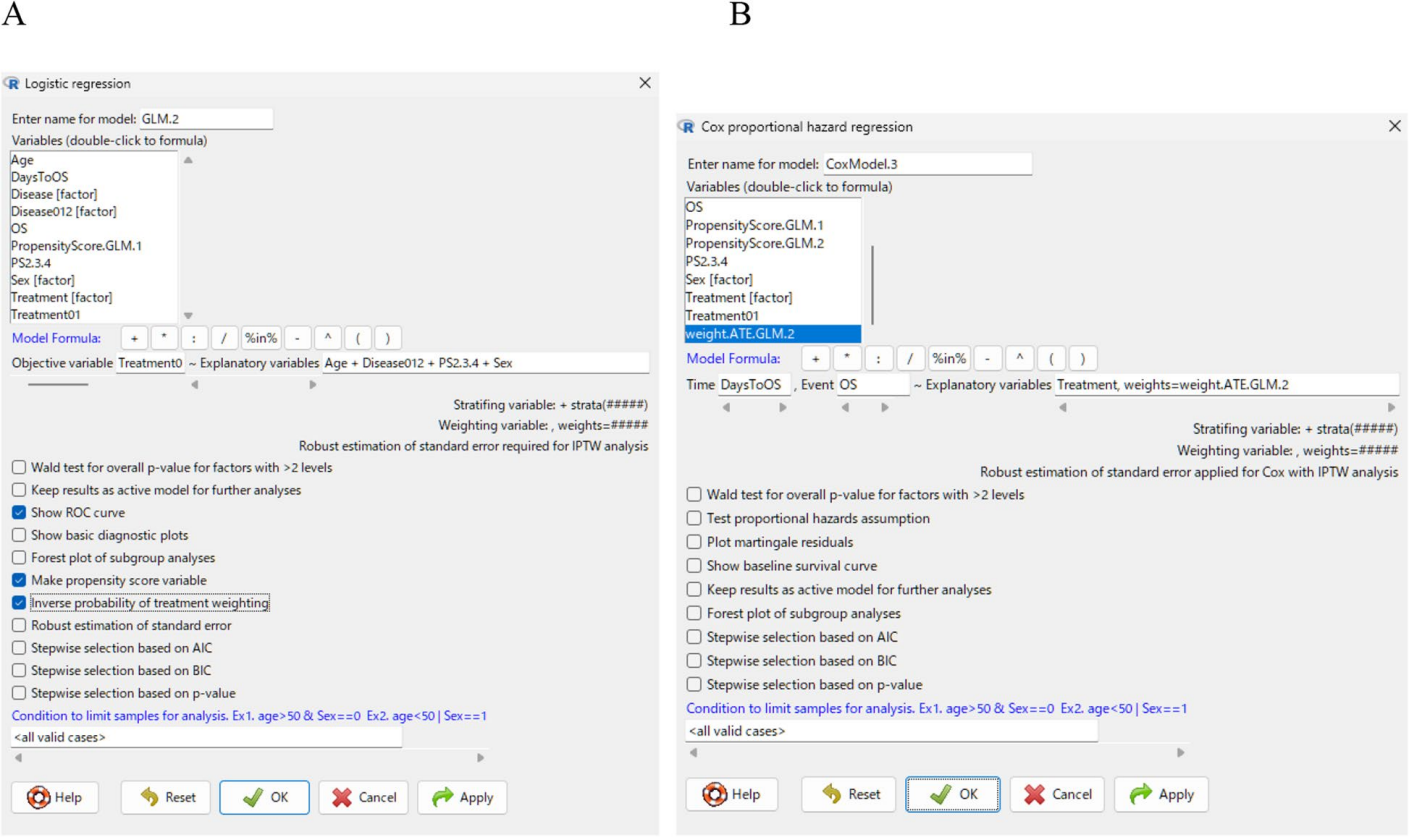
**A** Dialogue box for creating inverse probability weighting variables. **B** Dialogue box for performing Cox proportional hazards regression analysis using weighted variables

The variable for IPW can be created in the same way as the PS variable, except that “Disease012” should be selected instead of “Disease” in the “Explanatory variables,” and the option “Inverse probability of treatment weighting” should be checked (Fig. 4A). After this step, a weighting variable named “weight.ATE.GLM.X,” is created, and the standardized differences after weighting are displayed in the “Output” window. All four standardized difference values were reduced to less than 0.10 after weighting.

The weighting variable can then be used in a Cox proportional hazards regression analysis by selecting “Statistical analysis” > “Survival analysis” > “Cox proportional hazard regression”. In this procedure, “DaysToOS” should be specified as the “Time” variable, “OS” as the “Event” variable, and “Treatment, weights = weight.ATE.GLM.X” should be entered in the “Explanatory variables” field (Fig. 4B). The “Output” window shows the hazard ratio and its 95% confidence interval after weighting (Table 2C). In IPW analyses, confidence intervals should be calculated using a robust estimation of standard errors. The standard Cox proportional hazards regression function in EZR automatically applies this calculation; however, for logistic regression using IPW, “Robust estimation of standard error” must be selected.

### Final remarks

The results obtained from the conventional multivariate analysis and PS-based methods are summarized in Table 2. In this study, PS matching did not fully balance the patients’ background characteristics. The PS can also be applied for stratification or covariate adjustment, in addition to matching and IPW. Austin compared the performance of these methods in an extensive series of Monte Carlo simulations for time-to-event analyses [8] and reported that propensity score matching and IPW produced minimal bias, whereas stratification and covariate adjustment led to biased estimates of the hazard ratios. Therefore, matching or IPW is recommended for the PS analysis. However, PS methods are not inherently superior to conventional multivariate approaches; rather, they are particularly useful when the number of covariates requiring adjustment is large relative to the sample size [9]; however, they still require sufficient overlap between groups. In addition, PS analyses cannot replace randomized controlled trials, because these methods can only adjust for measured confounding factors.

## Data Availability

No datasets were generated or analysed during the current study.
